# Carboxymethyl Cellulose and Carboxymethyl Starch as Surface Modifiers and Greying Inhibitors in Washing of Cotton Fabrics

**DOI:** 10.3390/polym13071174

**Published:** 2021-04-06

**Authors:** Ksenija Višić, Tanja Pušić, Mirjana Čurlin

**Affiliations:** 1 Faculty of Textile Technology, University of Zagreb, Zagreb 10000, Croatia; ksenija.visic@ttf.unizg.hr; 2 Faculty of Food Technology and Biotechnology, University of Zagreb, Pierottijeva 6, Zagreb 10000, Croatia; mcurlin@pbf.hr

**Keywords:** cotton fabric, washing, CMC, CMS, zeta potential, deposits, whiteness, cluster analysis

## Abstract

This research is focused on cellulose and starch derivatives, carboxymethyl cellulose (CMC) and carboxymethyl starch (CMS), added to the detergent in washing reference cotton fabric in soft and hard water at 40, 60 and 90 °C. The applied polymers were analyzed through the potential of surface cellulose modification and inhibition of stain transfer from standard stain donors to modified and initial cotton fabrics. The surface modification of the cotton fabrics, characterized by the zeta potential and amounts of deposits, was coupled with the cluster analysis as well as a whiteness assessment. The obtained results of the zeta potential and degree of whiteness of the reference cotton fabrics before and after washing showed differences between CMC and CMS. The appropriateness of the cluster analysis was confirmed in assessing the potential of applied polymers for surface modification of cotton fabrics and greying inhibition.

## 1. Introduction

Washing removes dirt from textiles in an aqueous medium, with the synergy of four factors playing an important role: temperature, time, mechanical effect and chemical effect, which were combined into a process–theoretical system by Dr. Herbert Sinner in 1959 [1,2]. Water-soluble dirt and most detergent ingredients dissolve in water; the insoluble detergent ingredients and insoluble dirt are finely dispersed in water. With agitation, turbulence and the heating of water as a medium, the phenomenon of heat transfer and momentum affect the separating of stain particles from the fibers and their transfer to the bath. The retention of dirt on the free surface of the fibers is mostly caused by electrostatic interactions. To separate dirt particles, it is necessary to overcome the Van der Waals forces between the fiber and the particle, which is aided by repulsive electrostatic forces. In an aqueous medium, particles and textile fibers are negatively charged, and by adding an alkali, the negative charge of the fibers increase, but so does the charge of the particles, which favorably affects the washing effect [3,4]. Anionic surfactants adsorbed on textiles and particles further increase the negative charge and help disperse and keep particles in the bath. By creating a surface layer on the particles and grease droplets from removed stains, anionic surfactants act as stabilizers of the resulting dispersion and emulsion, which prevents re-deposition on the fiber. Deposition also prevents additional electronegative charging of particles and fibers, to which builders also contribute by softening the water and regulating pH values and thus improving the washing effect. Builders, based on ion exchange, combined with agents for complex binding of alkaline earth and heavy metal ions, can extract metal ions from dirt particles, which makes the particles porous and causes them to break the strength of binding to the fiber and the particle separates more easily. The action of the builder reduces the risk of interference of the surfactants [5]. In the presence of calcium and magnesium ions, detergents with soap form insoluble salts or soap scum, losing their detergency effect and, in addition, get deposited on surfaces forming incrustations. Textiles containing inorganic and organic deposits generated during washing have a grey tone, have an unpleasant odor, feel stiff and have an increased potential for skin irritation when worn [6,7]. Surfactants and builders cannot retain all the removed dirt during the washing in the bath. Thus, it is extremely important to improve the suspending and de-soiling powers by keeping the removed soils suspended during the aqueous phase and preventing their re-depositing on the textiles. Special polymers within a synergy with adequate surfactants, builders and enzyme cellulase are able to prevent the yellowing, the change in color and the greying of cotton textiles, and reduce the tendency of the fabric to deteriorate in appearance [8,9,10]. The yellowing and color change of washed textiles can also be affected when exposed to aging or finishing agents, e.g., softeners [11,12].

The polymer mostly used as a greying inhibitor or anti-redeposition agent (ARA) of cellulose textiles and is a derivative of cellulose—sodium carboxymethyl cellulose. Due to its water solubility, non-toxicity and biodegradability, carboxymethyl cellulose (CMC), as an anionic polyelectrolyte, is widely applied in numerous fields. In applications, most properties of CMCs depend on three parameters, namely, the molecular weight of the polymer, the average number of carboxymethyl substituents per anhydroglucose unit (or degree of substitution) and the distribution of carboxymethyl substituents along the polymer chain [13,14,15]. The degree of substitution of CMC for detergents varies from 0.6 to 0.9, which means that out of 10 anhydroglucose units, the carboxymethyl residue has 6 to 9 units [16,17,18].

The mechanism of action of this polymer is a specific adsorption on cellulose, specified as a very complex process determined by many factors and classified into three categories: (1) polymers, (2) cotton cellulose as an adsorbent, and (3) the composition and the properties of the solution [19].

These polymers have a specific orientation towards cellulose within several layers, which increases the number and density of negatively charged carboxyl groups and the negative charge of the textile surface.

In the first phase of rinsing, the dirt is removed, and then in the second phase, the connection between the greying inhibitor (ARA) and the fiber is broken. Modified starches with improved physicochemical properties relative to the native starch are also being researched as functional ingredients in numerous applications. Carboxymethyl starch (CMS), an etherified anionic starch derivative, is considered a green polymer with significant performance in the pharmaceutical, medical, cosmetics, food, environmental, and numerous other industries. This polymer has a low temperature of gelling, swelling and solubility in cold water and degree of substitution (DS), i.e., the number of hydroxyl groups replaced by carboxyl groups determines its properties [20,21].

In contrast to CMC, the properties of carboxymethyl starch (CMS) have not been investigated regarding surface modification and greying inhibition in the washing of cellulosic fibers. This is why this study is focused on comparing these two polymers, and their mixture (CMC + CMS) in surface modification and greying inhibition of cotton fabric. The polymers, CMC, CMS and their mixture, were added to a detergent dry slurry for washing the reference cotton fabrics in hard and soft water at 40, 60 and 90 °C.

The surface modification of the cotton fabric was analyzed after 10 washing cycles by determining the surface charge and deposit content relative to the initial one. The greying inhibition of the cotton fabric before and after modification, washed with stain donors, was analyzed by a whiteness measurement. Given the complexity of the washing process and the involvement of a large number of parameters, their impact was analyzed by cluster analysis as an advanced statistical technique that belongs to the field of multivariate statistical analysis (MVA).

## 2. Materials and Methods

### 2.1. Materials

Reference cotton fabric without fluorescent whitening agents and finishing agents (F), for which technical characteristics are prescribed by ISO 2267: 2001, was modified (MF) by adding the polymer–surface modifier (SM): CMC, CMS and their mixture, into a detergent dry slurry for 10 washing cycles in hard and soft water at temperatures of 40, 60 and 90 °C. The composition of the basic detergent, supplied by Labud d.d., Zagreb, Croatia is shown in Table 1.

The detergent contained anionic surfactant, soap, sodium percarbonate as bleach, silicate, builders (zeolites and sodium carbonate), fluorescent whitening agents and water. To this detergent, applied at a concentration of 5 g/L, surface modifiers CMC, CMS and their mixture (CMC + CMS) were added at a concentration of 0.4%. The supplier of powder CMC was Dencell, Bulgaria, and of the liquid CMS, Likval, Bosnia and Herzegovina.

The properties of CMC, CMS and their mixture in an inhibiting stain transfer were analyzed during a separate washing cycle of the reference cotton fabric (F) and the modified cotton fabric (MF), where EMPA stain donors (E-101, E-104, E-114 and E-116) were added to each individual fabric. The characteristics of these specific stained textile carriers or stain donors from the EMPA supplier (German: Eidgenössische Materialprüfungs und Forschungsanstalt, Swiss Federal Laboratories for Materials Science and Technology) are shown in Table 2.

The analysis of the potential of CMC, CMS polymers and their mixture in modifying cotton fabric through 10 cycles was performed in a laboratory washing machine with temperature variation (40, 60 and 90 °C) and water quality variation (hard (HW) and soft (SW)). The cycles were performed with a bath ratio (1:5), filling ratio (1:12), reversible rotation (40 to 50 rpm), and rinsing and cyclic centrifugation at 1400 rpm.

The degree of modification of the cotton fabrics (MF) under the described conditions of 10 washing cycles in relation to the reference cotton fabric (F) was analyzed by the streaming potential method and by determining the zeta potential as an appropriate method for analysis of the polymeric materials’ surface [22,23,24].

Through the amounts of deposits, the impact of water hardness and washing temperature on the polymer orientation on the surface of the cotton fabric and the degree of its modification (MF) in relation to the reference cotton fabric (F) was investigated.

The degree of inhibition of the stain transfer from the donor to reference cotton fabric (F) and modified fabric (MF) was evaluated before and after the 1st washing cycle in hard and soft water at 40, 60 and 90 ° by measuring the whiteness of the cotton fabric.

### 2.2. Methods

The degree of substitution (DS) affects the properties of polymers, and these must be determined before application [25]. The degree of substitution (DS) of the applied polymers (surface modifiers and greying inhibitors), CMC and CMS, was determined by the method IS 3520: 2004, Water-soluble sodium carboxymethyl cellulose.

The hardness of soft (SW) and hard water (HW), as an indicator of mineral load was determined by complexometric titration in an alkaline medium with the addition of indicator Eriochrom black T.

#### 2.2.1. Determination of Deposits

Deposits as incineration residues were determined directly on the standard reference cotton fabrics before (F) and after modification (MF). This residue comprises only a small fraction of the organic deposits; the amount of total ash depends on the magnitude of the mineral deposits according to ISO 4312: Surface active agents—Evaluation of certain effects of laundering—Methods of analysis and test for unsoiled cotton control cloth. The amount of ash from mineral substances is a more accurate index of the presence of mineral deposits, which originate from salts from the water or from the detergent, or their mutual interaction. The amount of total ash depends on the amount of mineral deposits, since only a small proportion of organic deposits is present in the total ash.

The incineration residue (A) of the reference cotton fabrics (F) and modified cotton fabric (MF) was determined according to the standard method (ISO 4312). Samples of reference cotton fabrics were incinerated in a muffle furnace for 2 h at 800 °C (Demiterm Easy 6, Eshterm, Croatia). The percentage of ash content was calculated from the masses of ash content and cotton fabric [26].

#### 2.2.2. Streaming Potential Method

The impact of surface modifiers (CMC, CMS, CMC + CMS) on the properties of cotton fabric (F) washed with base detergent in hard (HW) and soft water (SW) at temperatures of 40, 60 and 90 °C was analyzed via charge surfaces. The characterization of the charge of the cotton fabric surface before (F) and after modification (MF) was performed by the streaming potential method in an electrokinetic analyzer, EKA, A. Paar, Austria, where the cotton fabrics were the solid stationary phase, and the electrolyte solution KCl (1 mmol/l), the mobile phase. From the values of the streaming potential of the cotton fabric embedded in the adjustable gap cell (AGC) and the parameters in the system depending on the pH value of 1 mmol/L of KCl, the zeta potential (ζ) was calculated according to the Helmoltz–Smoluchovsky equation [27].

#### 2.2.3. Assessment of Whiteness

The deposition of stains removed from the reference donors on polymer modified fabrics (MF) and the initial fabric (F) in the 1st washing cycle was evaluated by the degree of their whiteness. Spectral parameters of reference fabrics, F, MF, F1 and MF1, were determined using the Spectrophotometer Data Color Spectraflash SF 600 Plus-CT, with the aperture size of 20 mm, under standard illumination D65, with the measuring range of 360–700 nm. The whiteness degree (W) was calculated automatically according to ISO 105-J02: 1997 Textiles—Tests for color fastness—Part J02: Instrumental assessment of relative whiteness and expressed as medium values of four individual measurements.

#### 2.2.4. Cluster Analysis

A cluster analysis was applied for evaluation of CMC, CMS and their mixture on deposits and the degree of inhibition of the stain transfer from donor to cotton fabrics before and after modification in washing with detergent in hard and soft water at 40, 60 and 90 °C.

This method belongs to the field of multivariate statistical analysis (MVA). Multivariate techniques are mainly empirical, and their task is to present the analysis of complex sets of data and their interrelationships in a simpler way, characterized by a lot of new information contained in the output data. They are applied in many areas of spectral data analysis (NIR, IR FTIR) [28] and time series models for monitoring parameters in environmental protection [29]. A cluster analysis is a method for dividing a group of objects into classes so that similar objects are in the same class. A cluster analysis searches for objects which are close together in the variable space [30]. The distance, *d*, between two points in *n* -dimensional space with coordinates *x_j_* and *y_j_* is usually taken as the Euclidean distance defined by Equation (1):

(1) $$d_{x,y}=\sqrt{\overset{J}{\underset{j=1}{\sum }}{\left(x_{j}-y_{j}\right)}^{2}}$$

A hierarchical cluster analysis is basically an iterative process of merging objects into groups. The method is based on the formation of clusters in a hierarchy, so that at each subsequent level, the number of clusters is decreased by one, with no possibility of transition from one group to another. This method is characterized by an unknown number of clusters determined on the basis of dendrograms. A dendrogram is a graphical presentation of the gradual combination of objects into clusters on which the distances between individual levels can be observed.

A single linkage method using TIBCO Statistics software to the incineration residues data, zeta potential data for deposit analysis and whiteness degree data for stain transfer were applied. As the measure of homogeneity, the error sum of squares (ESS) within the cluster was taken.

## 3. Discussion

The degree of substitution (DS) of surface modifiers and greying inhibitors, CMC and CMS, was determined. The obtained average values showed that the degree of substitution CMS (0.75) was higher than CMC (0.57).

Water hardness was analyzed, where the average value of hard water was expressed as concentration c (CaCO_3_) = 404.1 ppm and soft water concentration c (CaCO_3_) = 44.5 ppm.

### 3.1. Deposits: Incineration Residues (IR)

Given the binding mechanism of ARAs to cotton substrates, which act in synergy along with surfactants and builders during washing, it is necessary to consider the condition of the cotton fabric surface before and after washing in hard and soft water at 40, 60 and 90 °C. The content of deposits or residual substances is expressed as total ash, which primarily depends on the amount of mineral deposits, although in most cases this residue includes a small portion of organic deposits, since organic matter is not removed prior to incinerating the cotton cloth. The total ash of cotton fabric before washing is 0.2% and includes residues of mineral salts or mineral salts that have undergone certain chemical changes as a result of calcination and residue from incinerating organic salts. The builders in the formulation of the analyzed detergent are zeolite, in the ratio of 5.6% to 6.3%, and sodium carbonate, in the ratio of 35.0% to 40.0%. These builders have different mechanisms of binding alkaline earth elements from water: zeolite binds them by ion exchange and sodium carbonate by deposition; carbonates do not suspend soils in solution as zeolites do.

Through the content analysis of the deposits on the cotton fabric before (F) and after modification through 10 cycles (MF) in different conditions, the degree of surface loading with residues was determined. Since the obtained surface loading depends on a number of parameters of the washing process, the goal of this paper was to analyze the interaction of parameters that varied during the washing process. For the interrelationship of all parameters, a cluster analysis was performed on all the results of deposits (ash) of washed cotton fabrics (MF) with variations of detergent composition through different ARAs (CMC, CMS, CMC + CMS), and washing conditions (water (hard, soft) and temperature (40, 60 and 90 °C)).

**Figure 1:**
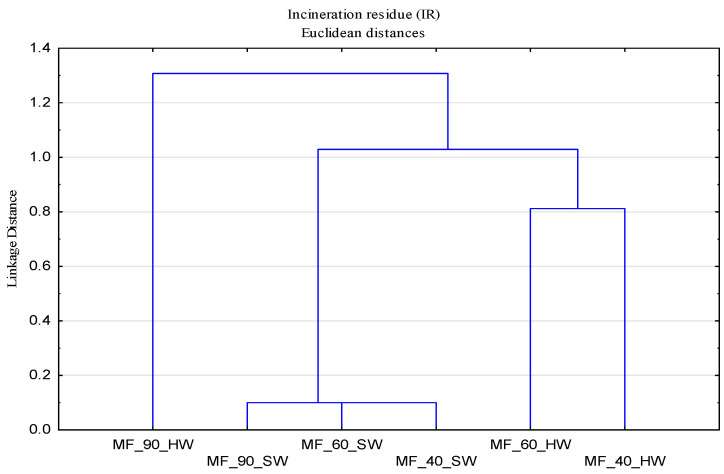
Dendrogram of the incineration residue data (IR) for carboxymethyl cellulose (CMC), carboxymethyl starch (CMS) and CMC + CMS as objects, and different temperatures, soft (SW) and hard water (HW) as variables.

A single linkage method to the incineration residues (IR) data was applied. The results are presented as dendrograms in Figure 1, which also illustrate the stages of the linkage. The vertical scale shows the Euclidean distance between the two groups at the point where they were combined.

The ash content of cotton fabrics modified in washing with detergent, in which the type of polymer was varied in soft water through 10 cycles at all temperatures, is almost identical. The presented dendrogram shows small differences in the stages of linkage of belonging to the group of samples washed in soft water. Washing in soft water shows no additional reactions or salt deposits on the fabric, since the value c (CaCO_3_) is low and the value of incineration residue can be compared with unwashed fabric, which is 0.2%.

The results of the cluster analysis also prove a considerable impact of temperature, with significant differences at 90 °C. From the dendrograms shown in Figure 1, it can be seen that the samples modified at 40 and 60 °C belong to the same group. There are obvious differences in the distribution of samples washed in hard water, where the impact of temperature and grouping of samples washed at 40 and 60 °C can be noticed, while samples washed at 90 °C belong to a separate group. The effect of water hardness is demonstrated through increased values of deposits on cotton fabrics modified by polymers in hard water compared to the same values on cotton fabrics modified in soft water. This can be attributed to the conditions of modification of the reference cotton fabric without stains through 10 washing cycles with detergent, the ingredients of which were not phenomenologically oriented for stain removal at the analyzed temperatures. Some organic components of detergent (soap and anionic surfactants) can, in interaction with alkaline earth elements, generate poorly soluble and/or insoluble deposits, which are deposited on the surface of the cotton fabric and increase the content of deposits expressed through total ash [31].

Since the washing process must be considered as a complex dispersion system in which interactions and synergistic effects of all the factors of the Sinner’s circle take place, and the impact of added surface modifiers must be defined, a cluster analysis of all samples was performed to group the samples according to surface modifier types. In view of the previously explained small impact of soft water as a medium, this analysis was performed only on samples washed in hard water.

**Figure 2:**
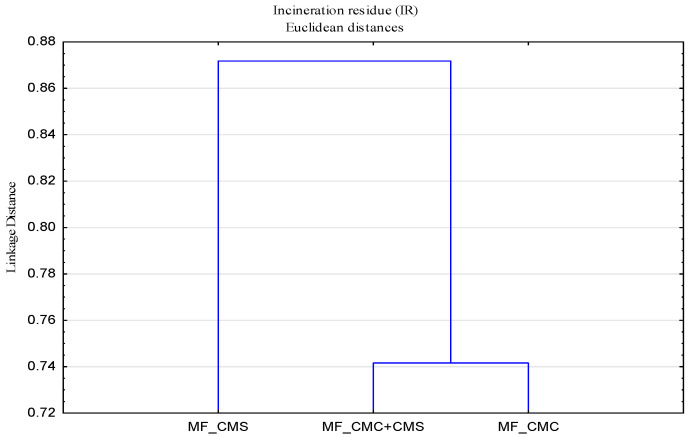
Dendrogram of incineration residue of modified cotton fabric (MF) as a function of surface modifiers in hard water.

From the obtained dendrograms shown in Figure 2, it can be observed that the polymer modifier of the CMS surface stands out, while CMC and the combination CMC + CMS belong to the same group, which indicates the significant impact and dominance of CMC in their mixture.

### 3.2. Zeta Potential of Reference Cotton Fabrics

**Figure 3:**
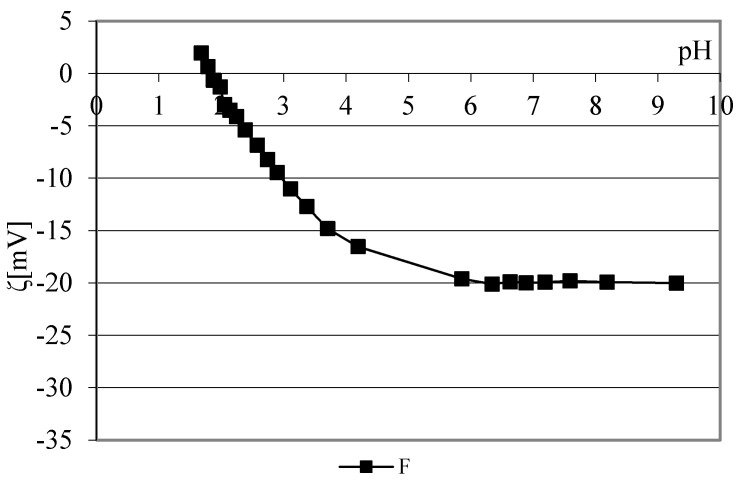
Zeta potential of reference cotton fabric (F) depending on pH of 1 mmol/L KCl.

The impact of polymer modifiers CMC, CMS and their mixture on the surface charge of the reference cotton fabric (F) after 10 cycles of washing (MF) with basic detergent was analyzed by titration curves of the zeta potential depending on the pH of 1 mmol/L KCl. The zeta potential of the reference cotton fabric (F) before modification depending on the pH of 1 mmol/L KCl is shown in Figure 3.

**Figure 4:**
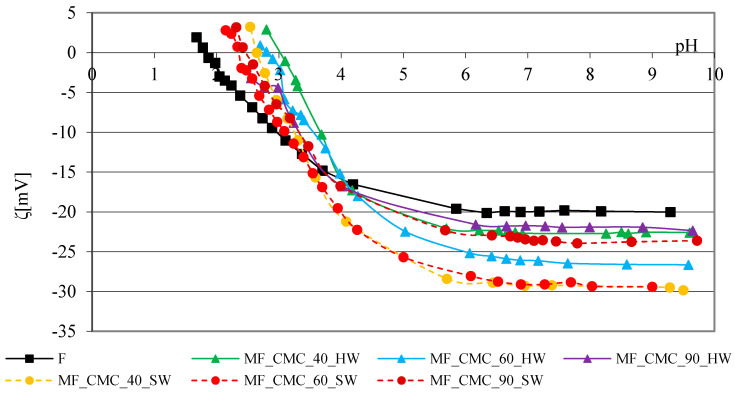
Zeta potential of cotton fabrics before and after 10 cycles of modification with detergent containing CMC in hard and soft water at 40, 60 and 90 °C depending on the pH of 1 mmol/L KCl.

**Figure 5:**
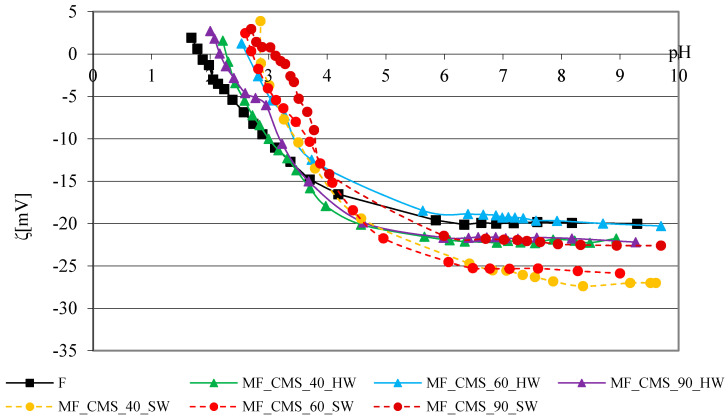
Zeta potential of cotton fabrics before and after 10 cycles of modification with detergent with CMS in hard and soft water at 40, 60 and 90 °C depending on the pH of 1 mmol/L KCl.

**Figure 6:**
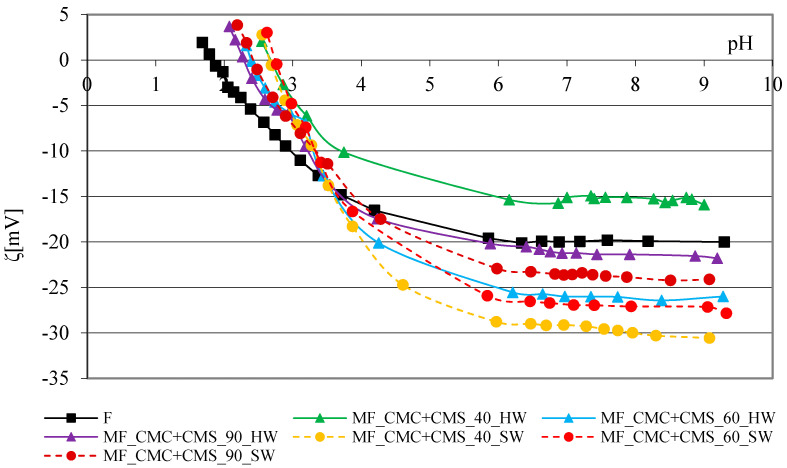
Zeta potential of cotton fabrics before and after 10 cycles of modification with detergent with CMC + CMS in hard and soft water at 40, 60 and 90 °C depending on the pH of 1 mmol/L KCl.

The zeta potential curve of the reference cotton fabric depending on the pH is characteristic of bleached cotton cellulose with hydroxyl (-OH) groups and a smaller number of carboxyl (-COOH) groups. The range of the curve from pH 9 to pH 6 is unchanged, and at lower pH values, it has a characteristic shape, which indicates the gradual neutralization of the negative charge up to reaching the isoelectric point [32]. The results of the zeta potential of cotton fabrics before and after adding modifiers, CMC, CMS and their mixture, in hard and soft water at 40, 60 and 90 °C depending on the pH of 1 mmol/L are shown graphically in Figure 4, Figure 5 and Figure 6. Figure 4 shows the results of the zeta potential of reference cotton fabrics before and after adding the CMC modifier to the basic detergent with variations of the water temperature and hardness.

The zeta potential values of the modified cotton fabrics (MF) under all the varied conditions in the range of pH 9 to pH 4 are more negative than the zeta potential values of the reference cotton fabric (F). Following the differences between the curves and the values, a behavior pattern cannot be established but generally, polymer-modified cotton fabrics in soft water have a more negative surface charge than polymer-modified fabrics in hard water. Based on the obtained ratios, CMC as a polymer impacts the increase of the negative charge of the cotton fabric surface modified in soft water at 40 and 60 °C. It agrees with the previous findings which showed the responsibility of CMC for the zeta potential and also the importance of electrostatic interactions in the adsorption process of CMC [33]. Cotton fabrics modified with CMC detergent at 90 °C in hard and soft water have a less negative surface charge compared to almost all the other polymer-modified cotton fabrics. This is linked with the impact of a higher temperature (90 °C) on the weaker potential of CMC, and not with the degree of surface loading with deposits, since their content on the surface of detergent-modified fabrics with CMC in hard water at 40, 60 and 90 °C is almost equal, and in soft water it is low.

The above ratio may also indicate some steric changes of fabric at 90 °C compared to 40 and 60 °C. According to the obtained results of the zeta potential of cotton fabrics modified with CMC at 40, 60 and 90 °C in hard water, and taking into account the surface condition in view of the content of deposits in the modification degree at 40 and 60 °C, the CMC orientation prevails, and at 90 °C the impact of temperature prevails.

Figure 5 shows the zeta potential curves of cotton fabrics before and after adding the surface modifier CMS to the basic detergent with temperature and water hardness variations.

The zeta potential values of all cotton fabrics modified with CMS are less negative than the values of cotton fabrics with CMC. The zeta potential curves of cotton fabrics modified with CMS in hard and soft water are characterized by grouping (1) soft water at 40 and 60 °C, and (2) hard water at 40 and 90 °C. The surface charge of the CMS-modified fabric in hard water at 60 °C is similar to the surface charge of the reference cotton fabric (F) in the highly alkaline range. Based on the obtained relations, it can be concluded that CMC as a modifier in the detergent leads to an increase in the negative charge of the cotton fabric surface in soft water at 40 and 60 °C. The curves in Figure 6 show the dependence of the zeta potential of cotton fabrics modified with a combination of polymers, CMC + CMS, at 40, 60 and 90 °C in soft and hard water, relative to the unwashed fabric.

**Figure 7:**
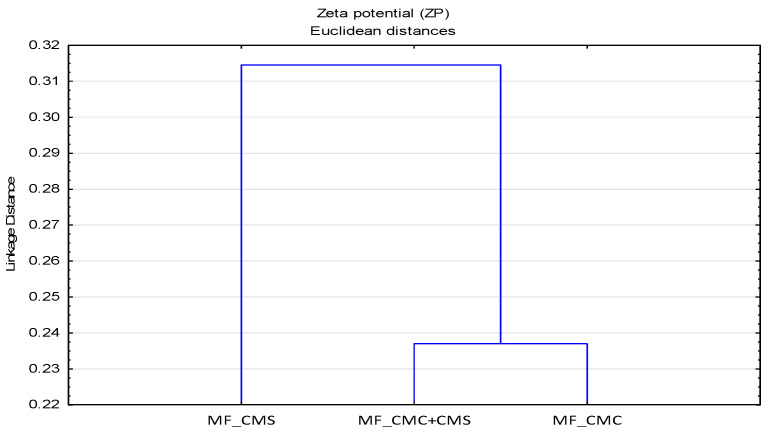
Dendrogram of zeta potential data of MF as a function of impact of surface modifiers (SMs).

The zeta potential curves are not grouped and are similar to the curves of fabrics modified by CMC, indicating a dominant impact of CMC over CMS in the CMC/CMS blend, which is shown by the grouping and lower surface charge of the modified cotton fabric. An interesting phenomenon in the modification of cotton fabric at 40 °C in hard water is reflected in the values of the zeta potential. The cotton fabric modified with CMC is more negative than the unmodified one, indicating a proper orientation of the -COOH polymer groups. A similar behavior is also observed by the modification with CMS. The specific orientation of the polymers, CMC and CMS, covered the cotton cellulose and prevented the dissociation of the reactive groups. This can be influenced by the rheological properties of the polymers. The cluster analysis and grouping of objects according to the action of SMs, i.e., the grouping of individual SMs for different values of pH 4, 6 and 9 was performed for the data on zeta potentials. On the dendrograms in Figure 7, the distribution by groups with isolated CMS can be observed, which represents the same distribution as in the IR results (Figure 2), but less linkage can be observed compared to the IR results.

**Figure 8:**
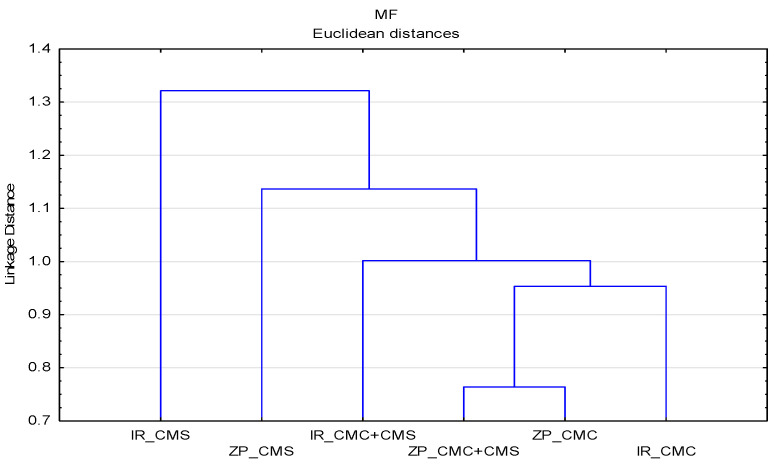
Dendrogram of the zeta potential (ZP) and incineration residue (IR) data of MF as a function of impact of SMs.

To understand the interaction of SMs in the determination of incineration residue (IR) and zeta potential (ZP), a cluster analysis was performed for all experimental IR and ZP data at pH 9, which represents the pH of the detergent solution. The results of the analysis are presented by dendrograms (Figure 8), which show a grouping of samples where CMC dominates up to the amount of the linkage distance of 1. After that, two separate groups were formed with samples subjected to the impact of CMS.

### 3.3. Whiteness Degree

**Figure 9:**
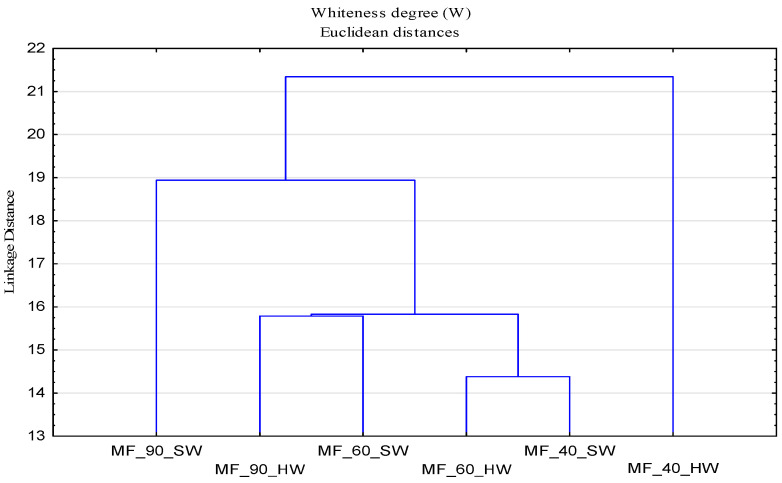
Dendrogram of the whiteness degree (W) as a function of temperature (T), soft (SW) and hard water (HW)**.**

The whiteness degree (W) of cotton fabrics after the 10th cycle modification with CMC, CMS and CMC + CMS (W_MF) added to the basic detergent is higher compared to that of standard pre-bleached cotton fabric (W_F), which has a low degree of whiteness, W = 45.90. To the basic detergent, stilbene-type fluorescent whitening agents for cellulosic fabrics and their mixture were added, which cumulatively increased the whiteness of all surface-modified cotton fabrics at all temperatures. Cotton fabrics modified at the temperature of 90 °C have the highest degree of whiteness. In this part of the research, related to the impact of modifiers (SMs), it is also important to examine the interaction of the parameters of the washing process related to temperature and washing in soft and hard water. For performing the cluster analysis, polymers modifiers were analyzed as objects, and temperature and soft and hard water media as variables. The results in the form of dendrograms are shown in Figure 9.

**Figure 10:**
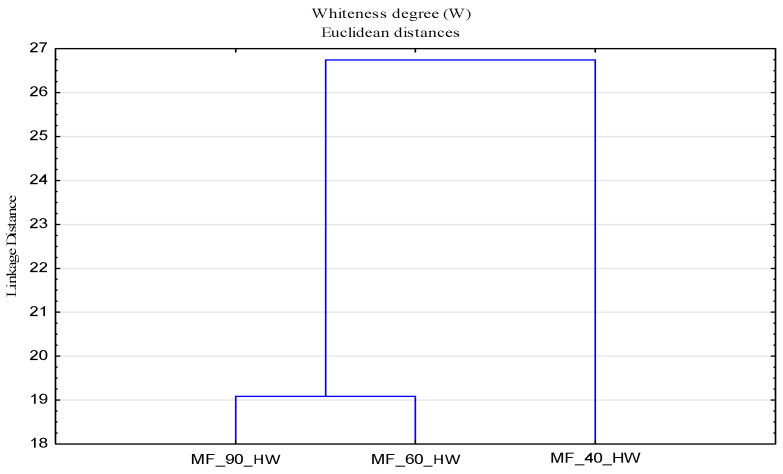
Dendrogram of the whiteness degree (W) of surface modified fabrics as a function of temperature (T) and hard water (HW).

**Figure 11:**
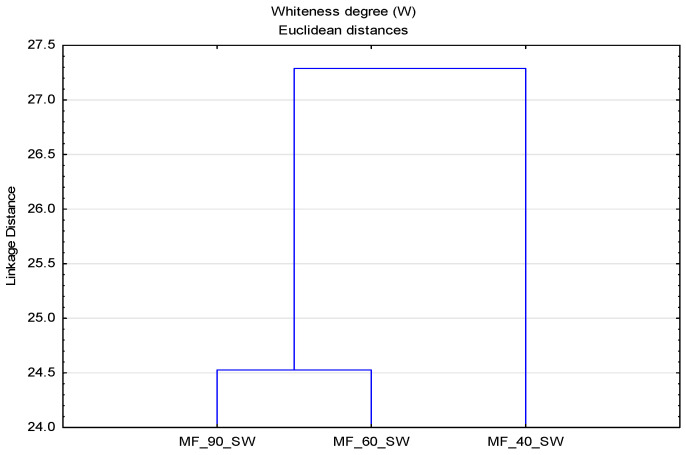
Dendrogram of the whiteness degree (W) of surface modified fabrics as a function of temperature (T) and soft water (SW).

In Figure 9, it can be observed that there is no clear distribution of temperature and media by groups, indicating a strong interaction of parameters, which requires a clear understanding of the washing process at different temperatures and the impact of deposits. The separation of samples modified at 40 °C in hard water and 90 °C in soft water required a further analysis. For a better assessment of the whiteness, an analysis was performed for surface modified samples in hard water (Figure 10) and soft water (Figure 11), by which a significant effect of higher temperatures on the whiteness of the modified fabric was proved.

**Figure 12:**
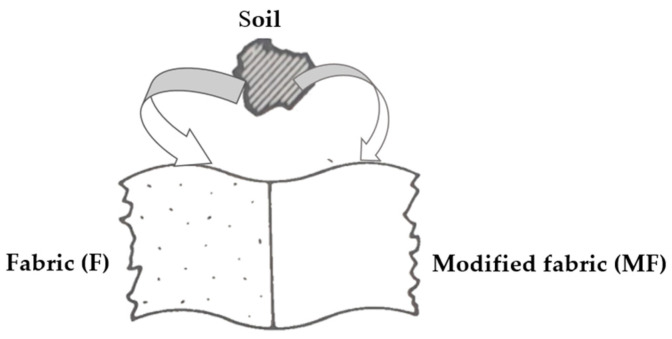
Schematic view of soil deposition on fabric and fabric modified with CMC/CMS.

The washing process of reference cotton fabrics (F) and modified fabrics (MF) was performed with stain donors to evaluate the action of greying inhibitors, CMC, CMS and their mixture. The degree of whiteness (W) of cotton fabrics was evaluated before and after modification (F and MF), and after the 1st cycle of washing (F1, MF1) of the cotton fabrics with stain donors. The potential of applied polymers in inhibiting soil deposition, i.e., their transfer from the bath to the clean reference and modified fabrics was assessed, Figure 12 [3].

The degree of inhibition of stain transfer, or re-deposition, is associated with certain types of stain donors (Table 2) that are classified into different groups: (1) pigment and oil (E-101 and E-104), (2) tannin (E-114), and (3) protein (E-116). The applied stain donors pigment in oil on the cotton and polyester/cotton textile carrier are more relevant in relation to tannins and proteins as donors. The power of removing the pigment in the oil represents the general washing effect and requires the synergy of all the factors of the Sinner’s circle. Tannins are removed by the physicochemical action of bleaches and their activators, where the washing temperature with the listed ingredients represents a significant factor. Protein stains are removed by the action of proteolytic enzymes, which is better achieved at lower temperatures [1,3].

The degree of whiteness of the reference cotton fabric washed with stain donors (F1) in all variations of washing conditions is higher than the whiteness of the reference cotton fabric (F, W = 45.90). The increase in whiteness of fabrics washed with basic detergent with CMC, CMS and CMC + CMS inhibitors at 40 °C is mild. The increase in the washing temperature from 40 to 60 °C typically increases the degree of whiteness of the washed fabric (F1) by about 20 units, while washing at the temperature of 90 °C significantly increases the whiteness of fabrics, which reaches values from 128.00 to 151.00. An improved whiteness effect is achieved in soft water for all baths. The whiteness of F1 cotton fabric confirms that there was no redeposition of soil from the bath on the F1 fabric. This cycle is dominated by the mechanism of the physical effect of the fluorescent whitening agent, especially at the washing temperature of 90 °C.

The degree of whiteness of cotton fabric modified with CMC, CMS, and CMC + CMS in washing with basic detergent at all temperatures is reduced in the subsequent washing cycle. This indicates the soil transfer from the bath to the surface of the cotton fabrics, through which the redeposition is partially inhibited. The results showed that the stain transfer is lower in soft water than in hard water. The reason for this is the more negative charge of the surface of the modified fabric due to the action of the polymer and the low amount of deposits on the surface. The largest decrease in whiteness of the modified cotton fabric (MF1), regardless of water hardness, was obtained in washing at 60 °C. In view of the previously defined significant impact of temperature on the whiteness degree (W) in the implemented protocol of modification and inhibition of stain transfer, a correlation matrix was set for individual modifiers and greying inhibitors for the whiteness of fabrics F1, MF and MF1. The correlation matrix presented in Table 3, Table 4 and Table 5 lists the values of Pearson coefficients that express the linear dependence of individual variables. The values can be expressed as negative (for an inversely proportional relationship) or positive (proportional relationship). The closer the value is to 1, the stronger the linear relationship. In Table 3, showing the correlations for CMC, only a significant correlation between temperature and whiteness for the F1 fabric can be observed. The whiteness of the modified MF and MF1 fabrics indicates no significant correlations neither with each other nor with the temperature. The correlations for CMS are shown in Table 4; significant correlations between temperature and F1, and temperature and MF fabric, as well as significant correlations between F1, MF and MF and MF1 are noticeable. The same correlations are evident for the polymer blend (CMC + CMS), which are shown in Table 5.

**Figure 13:**
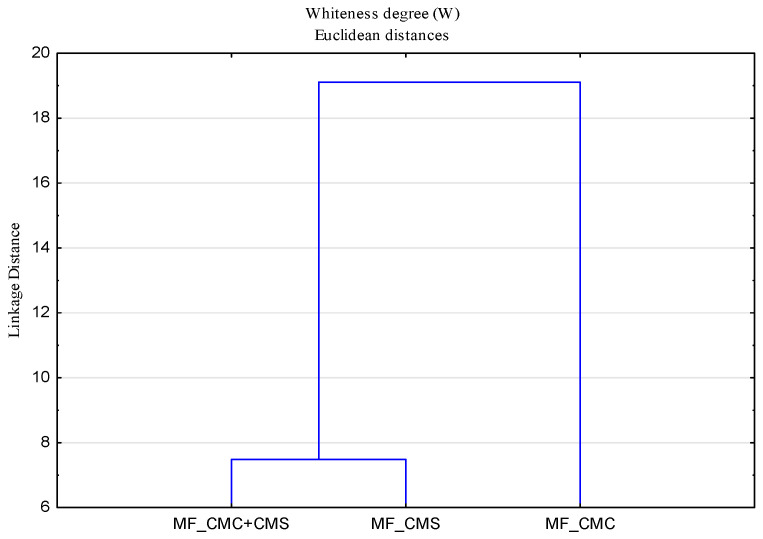
Dendrogram of the whiteness degree (W) of MF as a function of polymers surface modifiers.

**Figure 14:**
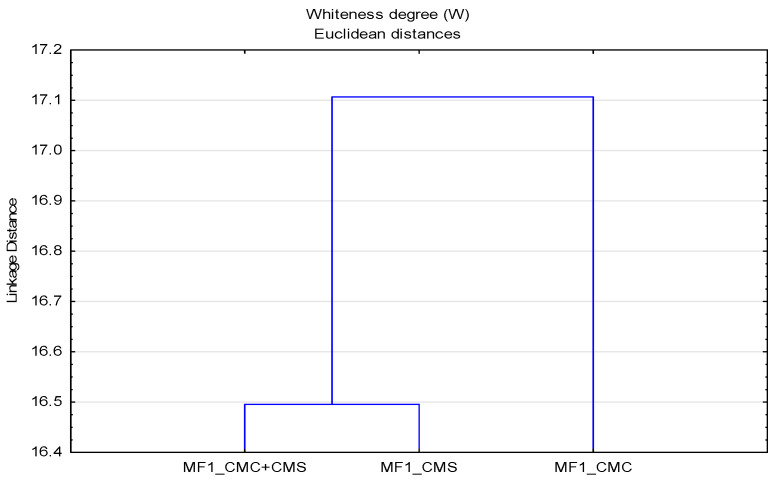
Dendrogram of the whiteness degree (W) of MF1 as a function of greying inhibition.

From the obtained results of the whiteness degree of cotton fabrics subjected to cycles of modification (MF) and washing (MF1) with changed parameters—bath composition (CMC, CMS, CMC + CMS), water (hard and soft), temperature (40, 60 and 90 °C)—a cluster analysis was performed, with the results shown by dendrograms in Figure 13 and Figure 14.

From the obtained results of the Pearson correlation parameters shown in Table 3, Table 4 and Table 5 and the groups shown in Figure 13 and Figure 14, significant differences can be observed in the application of CMC polymers in the protocol of modification and inhibition of stain transfer. Here, the dominant impact of CMS in the polymer blend can be highlighted, which is different from the previously presented results for IR and ZP, where CMC dominated the mixture.

## 4. Conclusions

The paper presents the application of cellulose and starch derivatives, carboxymethyl cellulose and carboxymethyl starch, in the processes of washing reference cotton fabric with detergent at temperatures of 40, 60 and 90 °C in two media: soft and hard water. The application and impact of the polymers were analyzed through the potential of surface modification of cotton cellulose and the greying inhibition of modified and initial cotton fabrics. The results of the surface modification, expressed as deposit and zeta potential, show a significant impact of CMC in the mixture of applied polymers CMC + CMS.

The results of greying inhibition expressed as whiteness show a more significant impact of CMS in the polymer mixture.

All of the obtained results indicate a substantial impact of temperature and water as a washing medium. Better insight into the individual impacts was gained by the cluster analysis. The cluster analysis has proven to be an effective tool for a better understanding and statistical confirmation of physicochemical interactions in the complex washing process with polymers.

Further research activities will be directed towards rheological properties of biopolymers in variation of concentrations and temperatures. Mechanical and advanced energy techniques in dynamic agitation will also be applied.

## Figures and Tables

**Table 1:** Composition of basic detergent.

Ingredient	w, %
Anionic surfactant	3.0
Soap	0.42–0.50
Sodium percarbonate	6.0
Zeolite	5.6–6.3
Silicates	5.1–5.7
Sodium carbonate	35.0–40.0
Stylbenic fluorescent whitening agent	0.3
Surface modifiers/ARA	-
Water	up to 100

**Table 2:** EMPA stain donors—standard stained fabrics (E-101, E-104, E-114 and E-116).

Label	Soil	Composition of Carrier	Mass per Unit Area, g/m^2^
E-101	carbon black/olive oil	cotton	90
E-104	carbon black/olive oil	polyester/cotton (65/35)	165
E-114	red wine	cotton	200
E-116	blood/milk/ink	cotton	200

**Table 3:** Correlation matrix of CMC.

Variable	Temperature	W_F1	W_MF	W_MF1
Temperature	1			
W_F1	**0.985**	1		
W_MF	0.725	0.626	1	
W_MF1	0.760	0.726	0.826	1

**Table 4:** Correlation matrix of CMS.

Variable	Temperature	W_F1	W_MF	W_MF1
Temperature	1			
W_F1	**0.97**	1		
W_MF	**0.869**	**0.862**	1	
W_MF1	0.745	0.782	**0.951**	1

**Table 5:** Correlation matrix of CMC + CMS.

Variable	Temperature	W_F1	W_MF	W_MF1
Temperature	1			
W_F1	**0.980**	1		
W_MF	**0.874**	**0.842**	1	
W_MF1	0.791	0.794	**0.927**	1

## Data Availability

The data presented in this study are available on request from the corresponding author.
